# Well-characterized sequence features of eukaryote genomes and implications for *ab initio* gene prediction

**DOI:** 10.1016/j.csbj.2016.07.002

**Published:** 2016-07-27

**Authors:** Ying Huang, Shi-Yi Chen, Feilong Deng

**Affiliations:** a Farm Animal Genetic Resources Exploration and Innovation Key Laboratory of Sichuan Province, Sichuan Agricultural University, Chengdu 611130, China; b College of Veterinary Medicine, Sichuan Agricultural University, Chengdu 611130, China

**Keywords:** Sequence features, Compositional properties, Functional signals, *Ab initio* gene prediction, Eukaryotes

## Abstract

*In silico* analysis of DNA sequences is an important area of computational biology in the post-genomic era. Over the past two decades, computational approaches for *ab initio* prediction of gene structure from genome sequence alone have largely facilitated our understanding on a variety of biological questions. Although the computational prediction of protein-coding genes has already been well-established, we are also facing challenges to robustly find the non-coding RNA genes, such as miRNA and lncRNA. Two main aspects of *ab initio* gene prediction include the computed values for describing sequence features and used algorithm for training the discriminant function, and by which different combinations are employed into various bioinformatic tools. Herein, we briefly review these well-characterized sequence features in eukaryote genomes and applications to *ab initio* gene prediction. The main purpose of this article is to provide an overview to beginners who aim to develop the related bioinformatic tools.

Due to tremendous progresses in terms of efficiency, accuracy and cost for the high-throughput sequencing technologies, a large number of genome sequences of eukaryotic, prokaryotic and archaea organisms are increasingly becoming available [1], [2]. These efforts are expected to open the window for better understanding the kinds of biological processes because essential information in principle is encoded in genome sequences. Nevertheless, it is also challenging for meaningfully decoding the huge amount of DNA sequences; for example, we are still infants in understanding biological implications of the substantial fraction sequences of “junk DNA” in eukaryote genomes, which don't encode any known proteins [3]. Additionally, a recent publication also revealed that the sequence context has functional consequences by influencing the substitution rate of adjacent nucleotides [4], which would complicate the biological explanation of genome sequences because the more complex mathematical models would be required.

By contrast to experimental investigations on biological functions, the *in silico* analysis of DNA sequences is essential in post-genomic era. There are many general properties of DNA sequence, such as GC content and base composition, having been well used for *in silico* analysis [5]. Additionally, *ab initio* prediction of gene structure is a critical step after sequencing whole genome and therefore has received much attention over the past decade [6]. Because of limitations of biological knowledge and bioinformatic algorithm, however, it still remains to be further improved on precision for these existing bioinformatic tools of gene prediction. In the present article, we briefly review these well-characterized features of DNA sequence and applications to *ab initio* gene prediction in eukaryotes. Although some literatures were published more than ten years ago, it is still helpful to provide an overall landscape for promoting the development of bioinformatic tools. Also, genome architectures for these available eukaryotic species are summarily illustrated in advance.

## 1 Outlines of genome architecture

To explore the evolutionary dynamics and biological consequences on genome size, base composition, and relative proportions of functional and nonfunctional sequences are deemed fascinating challenges in biology. The transposable genetic elements, in combination with natural selection, have been acknowledged to contribute to genome evolution, which result into considerable accumulation of repetitive sequences [7], [8], [9]. However, many proposed mechanisms trying to account for the genome evolution still remain uncertain or controversial, and these topics are also beyond scope of the present review. Fortunately, the recently prevailing approach of pan-genome analysis would be anticipated to provide more insights into this field [10].

**Fig. 1:**
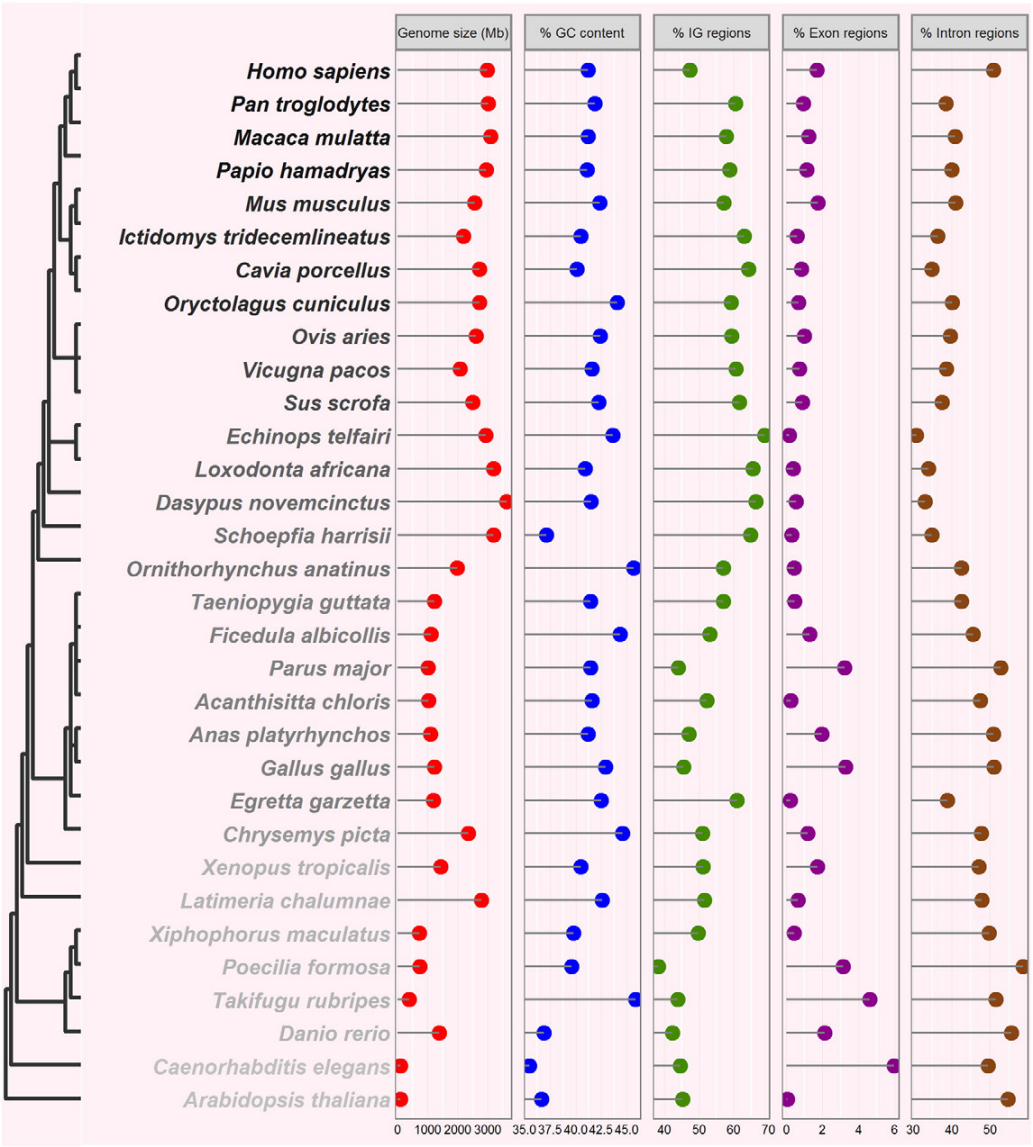
Architecture of eukaryotic genomes. A total of 32 representative species are included for comparatively illustrating the genome size, GC content, as well as respective proportions of intergenic regions (IG), exons and introns. In brief, all five indices were generated by the dissection of annotation information of reference genome (in GFF format) downloaded from NCBI (March, 2016); and these steps were performed using in-house scripts written in Python language. Additionally, the screenshot of NCBI taxonomic tree is employed to show the phylogenetic relationships among species, in which the full Latin scientific names of species were used.

According to intuitive expectation, the genome size would be proportional to species complexity, *i.e.*, the higher organisms have larger genomes. However, substantial variability of DNA content per haploid genome (C-value) have been widely observed even among the closely related species from same genus [3], which is thereby termed the C-value paradox. Scientific publications in eukaryotes on diversity patterns, evolutionary mechanisms and research methodologies in relation to genome size were recently summarized [11]. The traditional view suggests that more than 90% of human genome are nonfunctional and therefore regarded as “junk DNA”, whereas ENCODE project recently argued that up to 80% of genome sequences have functional roles [2], [12]. Of course, the two opinions are also being on the road for heated debate. Here, we analyzed the genome sequences for 32 representative eukaryote species and roughly illustrated their comparisons on genome size, GC content, and relative proportions of intergenic regions, exons and introns (Fig. 1). Unsurprisingly, an intuitional correlation between genome size and fraction of intergenic regions could be drawn out. Additionally, the proportions of exons and introns show consistent changes more or less.

## 2 Well-characterized features within genome sequence

**Fig. 2:**
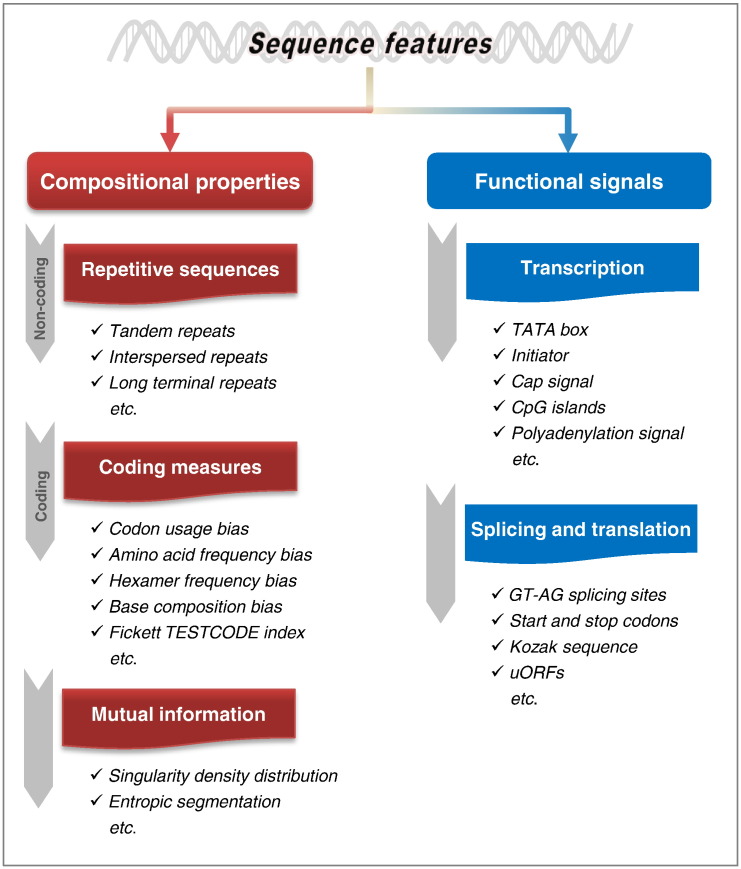
Schematic illustration of main sequence features.

Although it is impossible to be completely verified, the conserved features of DNA sequence would exist for corresponding to various biological functions, while some of them are already known but some unknown yet. On the basis of this supposition, we are able to perform *in silico* analysis of DNA sequences for functional investigations. On the whole, features of DNA sequence in eukaryotic genomes could be routinely categorized into two classes, including the compositional properties and functional signals (Fig. 2).

### 2.1 Compositional properties

#### 2.1.1 Repetitive sequences

Our knowledge on the organization of eukaryote genomes has dramatically increased due to ever-growing genome sequences [1]. A well-known feature of eukaryote genomes is that they consist of substantial proportion of repetitive sequences occurred in hundreds or thousands of times [13]. According to evolutionary origins and genomic distribution, repetitive DNA sequences could be overall classified into three types [14], [15], including the tandem repeats, interspersed repeats, and long terminal repeats (LTRs). Tandem repeats, such as microsatellites, minisatellites and satellites, are characterized by two or more contiguous repetitions of short fragments [16]. Interspersed repeats mainly include short and long interspersed elements; and both of them, together with LTRs, are evolutionarily derived from the transposable elements [17], [18]. As the evolutionary dynamics, diversity pattern, and biological function of repetitive sequences in eukaryote genomes have been intensively reviewed elsewhere [19], [20], [21].

The specific databases, such as Repbase Update [22] and SINEBase [23], provide platforms and computational tools for depositing, naming and annotating the repetitive sequences in eukaryotes. Meanwhile, various bioinformatic tools have been developed for finding repetitive sequences in genome, including RepeatMasker [24], PILER [25] and RepeatExplorer [26]. In human, it was estimated by *de novo* tool that about 70% of entire genome is repetitive or repeat-derived, which was higher than estimation using the alignment-based approaches [20], [27]. In practices, the repetitive sequences are always masked in advance for finding eukaryotic genes because of their absences for encoding proteins [28].

#### 2.1.2 Coding measures

Due to constraints of natural selection, base composition of protein-coding DNA sequences would significantly differ from non-coding sequences or random expectation. Various coding measures, in relation to base composition, had been early proposed with statistical virtue [29]. Among them, the most widely used measure is codon usage bias [30]; the observed frequencies for all 64 possible codons in a DNA sequence could be first counted. Alternatively, each codon could also be translated into amino acid and then generated the observed frequencies of 20 amino acids and stop codon. Subsequently, these observed frequencies of codons or amino acids are used to model the discriminant function for distinguishing coding from non-coding sequences. In more general way, the linguistic word in length of arbitrary *n* nucleotide acids can be phased and subjected to calculation of the observed frequencies. After comparing various word lengths, it has been acknowledged that the 6 bp word, which is also termed hexamer, would be the most informative index [31].

**Fig. 3:**
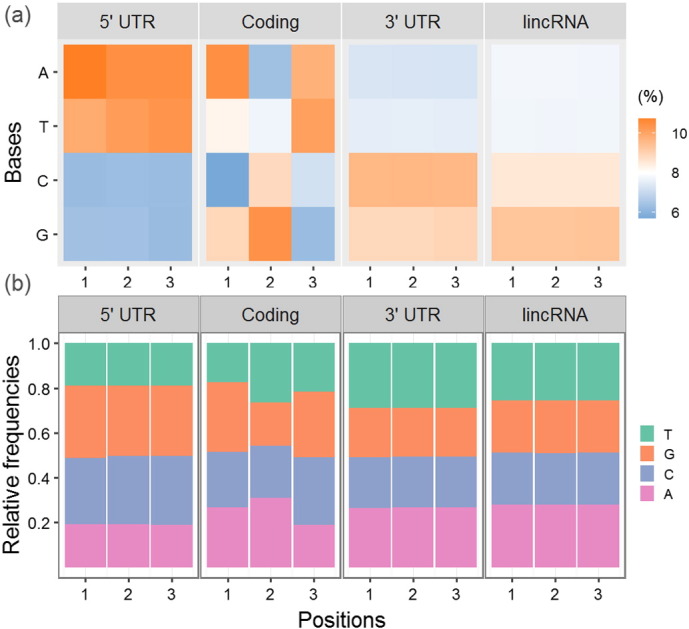
Base composition observed among three positions of coding codons or noncoding triplets. This analysis is totally based on 50, 909 reference sequences of mRNA and lincRNA in human. (a) The overall frequencies of nucleotide A, T, C and G among three positions are first computed for entire sequence. (b) The relative frequencies of four nucleotides at each position are further shown. For the non-coding sequences, the three-periodic nucleotide usage was calculated with arbitrary selection of start position.

Although genetic codon is represented as triplet, the degrees of biological conservation significantly differ among the first, second and third positions. Therefore, the base composition bias among three codon positions would be expected to provide valuable information for discriminating between coding and non-coding sequences [29]. To better demonstrate this issue, we analyzed the base frequencies among three positions between coding segment and untranslated regions for 38, 542 reference sequences of human mRNA. Additionally, 12, 367 sequences of known lincRNA in human were also included for comparison (Fig. 3). Our results clearly revealed the bias of base composition within coding segments in terms of both the absolute and relative frequencies. However, both intergenic and intron sequences should be further investigated. In fact, more than two decades ago, Fickett proposed a statistical index named Fickett TESTCODE [32], which combinationally utilized information of both base composition and codon usage bias and was employed for computationally estimating the coding potential of DNA sequence [33]. Recently, Python package of repDNA was published for efficiently generating feature vectors in relation to base composition of DNA sequences [5], which could facilitate analysis for biologists without well bioinformatic background.

#### 2.1.3 Other mutual information

Regardless of functional implications, it is also possible to find mutual information to discriminate between coding and non-coding sequences. For example, according to information-theoretic quantity, average mutual information was designed and taken as a species-independent statistical index for distinguishing coding from non-coding DNA sequences [34]. The segmentation method according to the estimated entropy in relation to base composition of DNA sequence was proven to be powerful for finding borders between coding and noncoding regions [35]. The local properties of DNA sequence, rather than global features, were also successfully used for partitioning the coding and non-coding regions in eukaryotic genome [36].

### 2.2 Functional signals

In addition to compositional properties of DNA sequences as mentioned above, genome sequences in eukaryotes would contain many intrinsic signals for guiding various biological functions, such as transcription, processing of pre-mRNA, and translation into amino acids [28]. Briefly, the well-known functional signals in relation to genic transcription mainly include TATA box, initiator, cap signal, CpG islands and polyadenylation signal. As for the genomic distribution, sequence characteristics and computational detection of transcriptional signals have been specifically addressed [37], [38], [39]. After being transcribed into pre-mRNA, splicing mechanism will be initiated for removing introns and producing mature mRNA; and during which splicing sites are recognized by the canonical presence of GT at donor site upstream of intron and AG at acceptor site downstream of introns, respectively [40], [41]. Beside start and stop codons, the Kozak sequence (GCC(A/G)CCAUGG) as well as upstream open reading frames (uORFs) would be the principal translational signals [42].

Although these functional signals would play important roles in predicting gene structure and organization, especially for protein-coding genes, two intrinsic limitations should be taken into account when including them into bioinformatic algorithm. First, there is no any statistical meaning by analyzing functional signals in DNA sequences. Second, not all of genes contain the canonical functional signals, *i.e.*, some signals would be completely absent or present by the non-canonical forms. For example, minor types of splicing sites have also been acknowledged in addition to canonical GT/AG [40]. In practices, therefore, both functional signals and compositional properties are always combined together for gene prediction.

## 3 Bioinformatic tools for *ab initio* gene prediction

Over past two decades, *ab initio* gene prediction from anonymous DNA sequences has acquired great achievements [43] and also boosted by need of genomic annotations when eukaryotic genomes become available [44]. For existing tools, much attention has been paid to prediction of protein-coding genes due to functional importance and algorithmic convenience. By contrast, the number and function of noncoding RNA (ncRNA) genes in eukaryotes, with exceptions of tRNAs and rRNAs, have remained largely unknown [45]. Therefore, the computational approaches for finding ncRNA genes in eukaryote genomes should be specifically addressed [46].

### 3.1 Brief description on prediction of protein-coding genes

The prevailing tools for computational prediction of protein-coding genes in eukaryotes have been considerably optimized, and on which specific reviews or comparatively technical analyses on their strengths and weaknesses had been already published [6], [47], [48], [49]. In the present review, therefore, we only summarize the pivotal features for these prevailing tools for *ab initio* prediction of eukaryotic genes in Table 1. Briefly, computational approaches of *ab initio* gene prediction could be discussed on two aspects, including the used information for describing DNA sequences and the employed algorithms for establishing the discriminant function. Various sequence features within eukaryote genomes in relation to gene prediction have been documented above. For modeling discriminant function, the most often used algorithms include Markov model and dynamic programming. Actually, most of them also utilize the information of sequence similarity by searching against database for improving prediction accuracy.

### 3.2 Prediction of ncRNA genes

Term of ncRNA generally refers to RNA molecule without needing to be translated into protein, which could directly function as RNA [56]. Therefore, ncRNAs would lack functional sense of ORFs and/or sequence features similar to protein-coding genes. However, absences of significant ORF or coding measures are not sufficient for supporting it is an ncRNA gene [3]. There are a variety of ncRNAs with differential structures and functions [45], [57], which significantly complicate *ab initio* prediction of ncRNA genes in eukaryote genome. In theory, a conserved feature for most if not all of ncRNAs is the presence of secondary structure, which would facilitate the computational prediction [46], [57].

#### 3.2.1 miRNA genes

The microRNA (miRNA) is an abundant family of ncRNAs playing ubiquitous roles for post-transcriptional regulations in eukaryotes with length of ~ 22 nucleotides. According to the biogenesis pathway, mature miRNAs are derived from intermediate precursor of pre-miRNAs in length of more than 70 nucleotides, which are almost characterized by a stem-loop structure [58], [59]. Another feature of miRNAs is highly evolutionary conserved on primary sequences and secondary structures even across taxonomically diverse species [45]. Therefore, the prevailing computational approaches for finding miRNA genes are preferable to simultaneously depend on both intrinsic sequence features and homology similarity [60], [61]. However, it is also necessary to predict the non-conserved or species-specific miRNA genes [62], hence we herein focus on *ab initio* approaches which completely utilize intrinsic features.

First, the potential to form hairpin structures is vital for selecting as candidates of miRNA genes, which could be computationally deduced on basis of the estimated free energy by tools of RNAfold [63] and Mfold [64]. Actually, the homology search-based approaches, such as MiRscan [65] and miRseeker [66], were also designed to first scan intergenic regions of entire genome and generate full list of candidates according to the deduced hairpin sequences before homology search. Therefore, design of the prevailing PalGrade tool is first to assign a score to each candidate sequence according to stability of computational hairpin, which, together with other features such as hairpin length and loop length, are subsequently used for establishing predictor of miRNA genes [62].

The support vector machine method can be used to discriminate between real and pseudo pre-miRNAs as implemented in triplet-SVM [67]. Similar to triplet-SVM, MiPred [68] additionally employed the thermodynamics-related features and random forest algorithm for achieving higher performance. A more sophisticated algorithm in ProMiR [69], termed the paired hidden Markov model-based probabilistic co-learning method, was proposed to utilize sequential and structural characteristics for efficiently predicting non-conserved miRNA genes. An alternative approach is HHMMiR, which used hierarchical hidden Markov model to describe the evolutionarily non-conserved hairpins [70]. A Naïve Bayes classifier (BayesmiRNAfind) was also proposed for prediction of miRNA genes, which efficiently utilize data from multiple species to provide better training dataset [71].

Recently, the speed of computational algorithm also began to be intentionally taken into consideration when predicting miRNA genes from entire genome. Tool of miRNAFold [72], an *ab initio* computational method, developed an approximation algorithm for searching hairpin sequences within genome and then resulted in significant decrease in number of candidates of interest. Along with rapid advances of high-throughput sequencing of small RNA, computational tools of miRNA prediction have been designed to utilize the sequenced short reads for structural analysis, such as MiRDeep and its varieties [73].

#### 3.2.2 lncRNA genes

Long noncoding RNAs (lncRNAs) are typically more than 200 nucleotides in length without protein-coding capability; and the estimated number in human genome would be significantly higher than protein-coding genes [74], [75]. Experimental examinations of lncRNA genes become feasible in eukaryotic species because they can be profiled by RNA-seq method due to their presences of poly(A) tails and other mRNA-like features [76]. In contrast to miRNAs, however, it is much difficult for *ab initio* predictions of genomic sequences which are transcripted into lncRNAs because of lack of informative features and evolutionary conservation [74]. Despite this fact, a few statistics of lncRNAs, such as the secondary structure, protein-coding potential and miRNA binding sites, have been proposed [77].

In practices, several existing tools could be used to computationally deduce the coding potential of cDNA sequences or the assembled transcripts from RNA-seq data. On basis of six biologically meaningful sequence features, including the possible ORFs and homology search hits, computational estimation of coding potential (CPC) was successfully established by support vector machine method [78]. Similar to CPC, computational tool of CPAT alternatively used the logistic regression method to model four sequence features for estimation of coding potential [33]. Of course, it is also expected to perform *ab initio* prediction of lncRNA genes from genome alone when our understanding on lncRNA biology significantly increase.

## 4 Concluding remarks

Along with the increasing sophistication and complexity of machine learning methods, it is anticipated that more and more biological processes could be computationally modeled. Meanwhile, the high-throughput sequencing technologies produce huge amounts of biological data each day, which would further motivate the development of computational biology. *Ab initio* computational prediction of eukaryotic genes, with a long history of intensive research, has considerably contributed to our understanding on the related biological questions. However, there still remain practical needs not only for further improvements in prediction accuracy of protein-coding genes but also for development of new approaches for finding ncRNA genes. In the present review, therefore, we outline the achievements in relation to two main aspects of *ab initio* gene prediction during the past two decades, including these well-characterized sequence features in eukaryote genomes and their practices in bioinformatic tools. However, the prediction methods on basis of homology search are not addressed here because of its relatively straightforward concept.

## Figures and Tables

**Table 1:** Summary of the selected tools for *ab initio* gene prediction in eukaryotes.

Tools	Years	Main sequence features	Algorithms
GeneID	1992 [50]	Splice sites; Start and stop codons; Coding signals	Rule-based system
GeneParser	1993 [51]	Splice site; Codon usage; Compositional complexity; Hexamer frequency; Length distribution; Periodic asymmetry	Dynamic programming
GENSCAN	1997 [52]	Coding signals; Length distributions and compositional features of exons, introns and intergenic regions	Generalized hidden Markov mode
HMMgene	1997 [53]	Coding, noncoding, and intergenic sequences	Hidden Markov model
Fgenesh	2000 [54]	Splice sites; Start and stop codons; Poly (A) signals; ORFs	Hidden Markov model
AUGUSTUS	2005 [55]	Sequences around splice sites, start and stop codons, and coding and non-coding regions; Length of exons, introns and intergenic regions	Generalized hidden Markov mode

Note: only these actively cited tools are included without subjective preference.

## References

[1] van Dijk E.L., Auger H., Jaszczyszyn Y., Thermes C. (2014). Ten years of next-generation sequencing technology. Trends Genet.

[2] Ellegren H. (2014). Genome sequencing and population genomics in non-model organisms. Trends Ecol Evol.

[3] Eddy S.R. (2012). The C-value paradox, junk DNA and ENCODE. Curr Biol.

[4] Aggarwala V., Voight B.F. (2016). An expanded sequence context model broadly explains variability in polymorphism levels across the human genome. Nat Genet.

[5] Liu B., Liu F., Fang L., Wang X., Chou K.-C. (2015). repDNA: a python package to generate various modes of feature vectors for DNA sequences by incorporating user-defined physicochemical properties and sequence-order effects. Bioinformatics.

[6] Sleator R.D. (2010). An overview of the current status of eukaryote gene prediction strategies. Gene.

[7] Fedoroff N.V. (2012). Transposable elements, epigenetics, and genome evolution. Science.

[8] Bennetzen J.L., Wang H. (2014). The contributions of transposable elements to the structure, function, and evolution of plant genomes. Annu Rev Plant Biol.

[9] Zhang G., Li C., Li Q., Li B., Larkin D.M. (2014). Comparative genomics reveals insights into avian genome evolution and adaptation. Science.

[10] Vernikos G., Medini D., Riley D.R., Tettelin H. (2015). Ten years of pan-genome analyses. Curr Opin Microbiol.

[11] Bainard J.D., Gregory T.R. (2013). Genome size evolution: patterns, mechanisms, and methodological advances. Genome.

[12] Consortium E.P. (2012). An integrated encyclopedia of DNA elements in the human genome. Nature.

[13] Charlesworth B., Sniegowski P., Stephan W. (1994). The evolutionary dynamics of repetitive DNA in eukaryotes. Nature.

[14] Steranka J., Valle D., Civin C., Wang T., Wheelan S. (2010). Mobile interspersed repeats are major structural variants in the human genome. Cell.

[15] López-Flores I., Garrido-Ramos M. (2012). The repetitive DNA content of eukaryotic genomes. Genome Dyn.

[16] Sonay T.B., Carvalho T., Robinson M., Greminger M., Krutzen M. (2015). Tandem repeat variation in human and great ape populations and its impact on gene expression divergence. Genome Res.

[17] Smit A.F. (1996). The origin of interspersed repeats in the human genome. Curr Opin Genet Dev.

[18] Smit A.F. (1999). Interspersed repeats and other mementos of transposable elements in mammalian genomes. Curr Opin Genet Dev.

[19] Jurka J., Kapitonov V.V., Kohany O., Jurka M.V. (2007). Repetitive sequences in complex genomes: structure and evolution. Annu Rev Genomics Hum Genet.

[20] Treangen T.J., Salzberg S.L. (2012). Repetitive DNA and next-generation sequencing: computational challenges and solutions. Nat Rev Genet.

[21] Biscotti M.A., Olmo E., Heslop-Harrison J.P. (2015). Repetitive DNA in eukaryotic genomes. Chromosome Res.

[22] Bao W., Kojima K.K., Kohany O. (2015). Repbase update, a database of repetitive elements in eukaryotic genomes. Mob DNA.

[23] Vassetzky N.S., Kramerov D.A. (2013). SINEBase: a database and tool for SINE analysis. Nucleic Acids Res.

[24] Smit A., Hubley R., G P (2015). RepeatMasker open-4.0.. http://www.repeatmasker.org.

[25] Edgar R.C., Myers E.W. (2005). PILER: identification and classification of genomic repeats. Bioinformatics.

[26] Novák P., Neumann P., Pech J., Steinhaisl J., Macas J. (2013). RepeatExplorer: a galaxy-based web server for genome-wide characterization of eukaryotic repetitive elements from next-generation sequence reads. Bioinformatics.

[27] de Koning A.J., Gu W., Castoe T.A., Batzer M.A., Pollock D.D. (2011). Repetitive elements may comprise over two-thirds of the human genome. PLoS Genet.

[28] Burge C.B., Karlin S. (1998). Finding the genes in genomic DNA. Curr Opin Biotechnol.

[29] Fickett J.W., Tung C.-S. (1992). Assessment of protein coding measures. Nucleic Acids Res.

[30] Moriyama E.N., Hartl D. (1993). Codon usage bias and base composition of nuclear genes in Drosophila. Genetics.

[31] Claverie J.-M. (1997). Computational methods for the identification of genes in vertebrate genomic sequences. Hum Mol Genet.

[32] Fickett J.W. (1982). Recognition of protein coding regions in DNA sequences. Nucleic Acids Res.

[33] Wang L., Park H.J., Dasari S., Wang S., Kocher J.-P. (2013). CPAT: coding-potential assessment tool using an alignment-free logistic regression model. Nucleic Acids Res.

[34] Grosse I., Herzel H., Buldyrev S.V., Stanley H.E. (2000). Species independence of mutual information in coding and noncoding DNA. Phys Rev E.

[35] Bernaola-Galván P., Grosse I., Carpena P., Oliver J.L., Román-Roldán R. (2000). Finding borders between coding and noncoding DNA regions by an entropic segmentation method. Phys Rev Lett.

[36] Kulkarni O.C., Vigneshwar R., Jayaraman V.K., Kulkarni B.D. (2005). Identification of coding and non-coding sequences using local Hölder exponent formalism. Bioinformatics.

[37] Down T.A., Hubbard T.J. (2002). Computational detection and location of transcription start sites in mammalian genomic DNA. Genome Res.

[38] Heintzman N.D., Stuart R.K., Hon G., Fu Y., Ching C.W. (2007). Distinct and predictive chromatin signatures of transcriptional promoters and enhancers in the human genome. Nat Genet.

[39] Shahmuradov I.A., Solovyev V.V. (2015). Nsite, NsiteH and NsiteM computer tools for studying transcription regulatory elements. Bioinformatics.

[40] Sheth N., Roca X., Hastings M.L., Roeder T., Krainer A.R. (2006). Comprehensive splice-site analysis using comparative genomics. Nucleic Acids Res.

[41] Fox-Walsh K.L., Hertel K.J. (2009). Splice-site pairing is an intrinsically high fidelity process. Proc Natl Acad Sci U S A.

[42] Jackson R.J., Hellen C.U., Pestova T.V. (2010). The mechanism of eukaryotic translation initiation and principles of its regulation. Nat Rev Mol Cell Biol.

[43] Fickett J.W. (1996). Finding genes by computer: the state of the art. Trends Genet.

[44] Consortium I.H.G.S. (2004). Finishing the euchromatic sequence of the human genome. Nature.

[45] Cech T.R., Steitz J.A. (2014). The noncoding RNA revolution—trashing old rules to forge new ones. Cell.

[46] Eddy S.R. (2002). Computational genomics of noncoding RNA genes. Cell.

[47] Mathé C., Sagot M.F., Schiex T., Rouzé P. (2002). Current methods of gene prediction, their strengths and weaknesses. Nucleic Acids Res.

[48] Wang Z., Chen Y., Li Y. (2004). A brief review of computational gene prediction methods. Genomics Proteomics Bioinformatics.

[49] Goel N., Singh S., Aseri T.C. (2013). A comparative analysis of soft computing techniques for gene prediction. Anal Biochem.

[50] Guigó R., Knudsen S., Drake N., Smith T. (1992). Prediction of gene structure. J Mol Biol.

[51] Snyder E.E., Stormo G.D. (1993). Identification of coding regions in genomic DNA sequences: an application of dynamic programming and neural networks. Nucleic Acids Res.

[52] Burge C., Karlin S. (1997). Prediction of complete gene structures in human genomic DNA. J Mol Biol.

[53] Krogh A. (1997). Two methods for improving performance of an HMM and their application for gene finding. Genome Res.

[54] Salamov A.A., Solovyev V.V. (2000). Ab initio gene finding in Drosophila genomic DNA. Genome Res.

[55] Stanke M., Morgenstern B. (2005). AUGUSTUS: a web server for gene prediction in eukaryotes that allows user-defined constraints. Nucleic Acids Res.

[56] Mattick J.S., Makunin I.V. (2006). Non-coding RNA. Hum Mol Genet.

[57] Nawrocki E.P., Burge S.W., Bateman A., Daub J., Eberhardt R.Y. (2014). Rfam 12.0: updates to the RNA families database. Nucleic Acids Res.

[58] Ambros V. (2001). microRNAs: tiny regulators with great potential. Cell.

[59] Chong M.M., Zhang G., Cheloufi S., Neubert T.A., Hannon G.J. (2010). Canonical and alternate functions of the microRNA biogenesis machinery. Genes Dev.

[60] Mendes N., Freitas A.T., Sagot M.-F. (2009). Current tools for the identification of miRNA genes and their targets. Nucleic Acids Res.

[61] Gomes C.P., Cho J.-H., Hood L., Franco O.L., Pereira R.W. (2013). A review of computational tools in microRNA discovery. Front Genet.

[62] Bentwich I., Avniel A., Karov Y., Aharonov R., Gilad S. (2005). Identification of hundreds of conserved and nonconserved human microRNAs. Nat Genet.

[63] Hofacker I.L., Fontana W., Stadler P.F., Bonhoeffer L.S., Tacker M. (1994).

[64] Zuker M. (2003). Mfold web server for nucleic acid folding and hybridization prediction. Nucleic Acids Res.

[65] Lim L.P., Glasner M.E., Yekta S., Burge C.B., Bartel D.P. (2003). Vertebrate microRNA genes. Science.

[66] Lai E.C., Tomancak P., Williams R.W., Rubin G.M. (2003). Computational identification of Drosophila microRNA genes. Genome Biol.

[67] Xue C., Li F., He T., Liu G.-P., Li Y. (2005). Classification of real and pseudo microRNA precursors using local structure-sequence features and support vector machine. BMC Bioinformatics.

[68] Jiang P., Wu H., Wang W., Ma W., Sun X. (2007). MiPred: classification of real and pseudo microRNA precursors using random forest prediction model with combined features. Nucleic Acids Res.

[69] Nam J.-W., Shin K.-R., Han J., Lee Y., Kim V.N. (2005). Human microRNA prediction through a probabilistic co-learning model of sequence and structure. Nucleic Acids Res.

[70] Kadri S., Hinman V., Benos P.V. (2009). HHMMiR: efficient de novo prediction of microRNAs using hierarchical hidden Markov models. BMC Bioinformatics.

[71] Yousef M., Nebozhyn M., Shatkay H., Kanterakis S., Showe L.C. (2006). Combining multi-species genomic data for microRNA identification using a Naive Bayes classifier. Bioinformatics.

[72] Tempel S., Tahi F. (2012). A fast ab-initio method for predicting miRNA precursors in genomes. Nucleic Acids Res.

[73] Friedländer M.R., Chen W., Adamidi C., Maaskola J., Einspanier R. (2008). Discovering microRNAs from deep sequencing data using miRDeep. Nat Biotechnol.

[74] Ponting C.P., Oliver P.L., Reik W. (2009). Evolution and functions of long noncoding RNAs. Cell.

[75] Quek X.C., Thomson D.W., Maag J.L., Bartonicek N., Signal B. (2015). lncRNAdb v2. 0: expanding the reference database for functional long noncoding RNAs. Nucleic Acids Res.

[76] Necsulea A., Soumillon M., Warnefors M., Liechti A., Daish T. (2014). The evolution of lncRNA repertoires and expression patterns in tetrapods. Nature.

[77] Volders P.J., Verheggen K., Menschaert G., Vandepoele K., Martens L. (2015). An update on LNCipedia: a database for annotated human lncRNA sequences. Nucleic Acids Res.

[78] Kong L., Zhang Y., Ye Z.-Q., Liu X.-Q., Zhao S.-Q. (2007). CPC: assess the protein-coding potential of transcripts using sequence features and support vector machine. Nucleic Acids Res.

